# A Mild and Facile Synthesis of Amino Functionalized CoFe_2_O_4_@SiO_2_ for Hg(II) Removal

**DOI:** 10.3390/nano8090673

**Published:** 2018-08-29

**Authors:** Xi Wang, Zhenzong Zhang, Yuhao Zhao, Kai Xia, Yongfu Guo, Zan Qu, Renbi Bai

**Affiliations:** 1 Center for Separation and Purification Materials &Technologies, Suzhou University of Science and Technology, Suzhou 215009, China; 1613022027@post.usts.edu.cn (X.W.); 1713022013@post.usts.edu.cn (Z.Z.); 1713022014@post.usts.edu.cn (Y.Z.); 1713022009@post.usts.edu.cn (K.X.); ceebairb@live.com (R.B.); 2 Jiangsu Provincial Key Laboratory of Environmental Science and Engineering, Suzhou University of Science and Technology, Suzhou 215009, China; quzan@sjtu.edu.cn; 3 School of Environmental Science and Engineering, Shanghai Jiao Tong University, Shanghai 200240, China

**Keywords:** magnetic materials, amino functionalization, adsorption, heavy metals, mercury

## Abstract

To avoid the dangerous operational conditions, shorten the preparation time, and improve the adsorption performance of amino-functionalized nanomagnetic materials with a core–shell structure, a magnetic nanocomposite of CoFe_2_O_4_@SiO_2_ was successfully functionalized with amino group (−NH_2_) through a mild and facile hydrothermal method without the use of any toxic or harmful solvents at a relatively low temperature. The preparation time of the key steps of amino functionalization was shortened from 30 h to about 10 h. The core-shell structure and successful grafting were confirmed by various means. The amino-functionalized CoFe_2_O_4_@SiO_2_ was used for the removal mercury (Hg(II)), a heavy metal, and exhibited excellent magnetic properties and a high Langmuir adsorption capacity of 149.3 mg Hg(II)/g. The adsorption of Hg(II) onto CoFe_2_O_4_@SiO_2_–NH_2_ followed the pseudo-second-order kinetic equation and Langmuir model. The thermodynamic data showed that the Hg(II) adsorption process was achieved through spontaneous exothermic and monolayer adsorption with electrostatic adsorption and chemisorption. In addition, the as-prepared CoFe_2_O_4_@SiO_2_–NH_2_ nanoparticles had a good reusable value, good application performance and stability, and can provide a mild and facile way to remove heavy metals from aqueous solution.

## 1. Introduction

Mercury (Hg) is one of the most toxic heavy metals in the water environment. It has received extensive attention because of its non-biodegradability and easy enrichment in the biological chain [1]. Mercury generally exists in two forms: as elemental (Hg^0^) [2] and ionic mercury (Hg^2+^) [3,4]. Between them, the most toxic is Hg^2+^.

Nowadays, more and more mercury pollution problems are receiving international and extensive attention. For the safety of people, governments designate plans to eliminate mercury pollution, and the Chinese government has released corresponding regulations, including “Power Plant Air Pollutants Emission Standards” (GB 13223-2011) and the “Emission Standard of Pollutants for Electroplating” (GB 21900-2008). A series of global and national policy activities have also been implemented to reduce mercury pollution in the environment [5,6].

In recent years, modified magnetic nanomaterials, used as promising environmentally friendly absorbents, have been very effective in separating heavy metal pollutants from water phases [7,8,9]. This recyclability makes them easy to regenerate, indicating that they are as green and sustainable as other adsorbents [10,11,12].

Among these magnetic nanomaterials (MNPs), CoFe_2_O_4_ MNPs have attracted much attention because of their unique physical and chemical capabilities, such as their high coercivity, magnetization, and mechanical hardness [13,14]. These characteristics make CoFe_2_O_4_ MNPs ideal magnetic core carriers [15]. However, CoFe_2_O_4_-based core-shell carriers have little adsorption capacity for heavy metals [16] and usually have to be modified on their surface to obtain a high adsorption capacity. Common grafting methods are to coat CoFe_2_O_4_ particles with a packing layer and then graft onto its outer surface with amino group (−NH_2_), thiol group (−SH), sulfonic group (−HSO_3_), phosphoric acid, and other functional groups [9,14,17].

However, most grafting methods of magnetic nanoadsorbents with a core–shell structure are too complicated, and almost all of these adsorbents use toxic, harmful, or hazardous solvents as a reaction media, such as o-xylene [18], acetone [19], toluene [3], and hexane [20]. Furthermore, in the case of the unavoidable use of organic solvents, the recovery of these solvents requires the use of fractional liquid extraction, adsorption, fractionation, and other methods, which greatly increases the cost of the experiment, and the possibility of secondary pollution to the environment [12,21,22,23].

Meanwhile, there are other grafting experiments which take place under water-alcohol system, but nitrogen protection and a high temperature are still required during the process of amino functionalization [24]. In addition, the whole operational time is relatively long, usually over 30 h. These defects greatly limit the practical application of amino functionalization. 

Therefore, this study aims to explore a simple, mild, and facile method to prepare core-shell CoFe_2_O_4_-based adsorbents with −NH_2_ as the grafted group, and meanwhile, enhance the adsorption capacity for heavy metal ions. In the present article, the amino-functionalized magnetic CoFe_2_O_4_@SiO_2_–NH_2_ nanoparticles with a core–shell structure were synthesized with tetraethyl silicate (TEOS) and 3-Aminoprppyltriethoxysilane (APTES) under relatively mild conditions. The as-prepared composite adsorbent of CoFe_2_O_4_@SiO_2_–NH_2_ was employed to remove Hg(II) ions from aqueous solution. In addition, adsorption factors like optimum pH, initial concentration of Hg(II), real application, stability, adsorption kinetics, isotherm, and thermodynamics were studied. Finally, the possible mechanism was also proposed.

## 2. Materials and Experiment Methods

### 2.1. Chemicals and Materials

3-Aminopropyltriethoxysilane (APTES, 99.0%), tetraethyl silicate (TEOS), ethylene glycol (EG), ethanol alcohol absolute, ferric chloride (FeCl_3_·6H_2_O), cobalt chloride (CoCl_2_·6H_2_O), anhydrous sodium acetate (CH_3_COONa), polyethylene glycol (4000), and ammonia (NH_3_·H_2_O, 25–28 wt.%) were purchased from Macklin Reagent (Shanghai, China). All the reagents were analytical grade.

### 2.2. Preparation of CoFe_2_O_4_ MNPs

CoFe_2_O_4_ MNPs were synthesized using hydrothermal technology. Typically, FeCl_3_·6H_2_O (5.405 g) and CoCl_2_·6H_2_O (2.375 g) were dissolved in ethylene glycol (90 mL), and stirred at 323 K for 30 min. Then, a certain amount of CH_3_COONa (7.335 g) and polyethylene glycol (2.0 g) were added into the mixture with magnetic stirring for 30 min. Subsequently, the brown viscous liquid was moved into a Teflon reactor (150 mL) and placed in an oven at 473 K for 16 h without stirring. After that, magnetic decanting was used to wash with ethanol three to four times, the above solution was then further washed with ultrapure water until the pH was close to neutral. The obtained CoFe_2_O_4_ materials were dried and bagged for use.

### 2.3. Preparation of CoFe_2_O_4_@SiO_2_ MNPs

CoFe_2_O_4_ MNPs (0.30 g) were dispersed in a mixed solution containing pure water (30 mL) and anhydrous ethanol (120 mL), and submitted to mechanical stirring after 1 h of ultrasonic dispersion. NH_3_·H_2_O (1 mL) was added and reacted for 15 min under mechanical stirring (200 rpm) and heating conditions. Then TEOS solution (2 mL) was evenly dropped to react for 8 h after the solution temperature reached 313 K. Finally, the above prepared brown mixture was washed and dried at 313 K.

### 2.4. Preparation of CoFe_2_O_4_@SiO_2_–NH_2_ MNPs

Preparation of solution A: The above-prepared material CoFe_2_O_4_@SiO_2_ MNPs (0.3 g) was added into a mixed solution containing pure water (20 mL) and anhydrous ethanol (80 mL), subjected to ultrasonic dispersion until the material uniformly dispersed in the solution. Preparation of solution B: TEOS (0.75 mL) was dissolved into ethanol solution (20 mL) with ultrasonic treatment for half an hour. Preparation of Solution C: APTES (0.75 mL) was dissolved into ethanol solution (20 mL) with ultrasonic treatment for half an hour.

**Figure 1:**
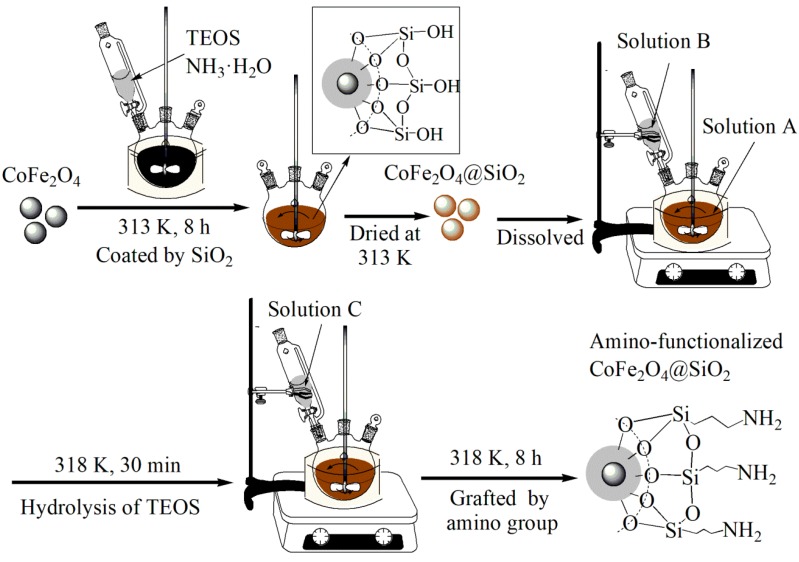
Synthesized schematic diagram of CoFe_2_O_4_@SiO_2_–NH_2_ and silanization reaction between silica and 3-aminopropyltriethoxysilane (APTES).

Figure 1 shows the synthesis schematic diagram of CoFe_2_O_4_@SiO_2_–NH_2_ MNPs. The formation of a SiO_2_ shell outside CoFe_2_O_4_ MNPs was realized via the addition of TEOS. Firstly, CoFe_2_O_4_ MNPs were dispersed in a mixed solution with mechanical stirring (200 rpm) at 313 K for 8 h. Because of the hydrolysis of TEOS, abundant Si–OH groups were formed. Subsequently, the as-obtained Si–OH reacted with Fe–OH groups on the surface of CoFe_2_O_4_ MNPs to form a silicon shell with –OH groups outside CoFe_2_O_4_. In order to quantify the amount of additive APTES, solution A was formed as mentioned above. Subsequently, solution B was added dropwise into solution A. The stirring was continued for half an hour after the dropwise addition of solution B. 

Afterwards, solution C was added dropwise into the above mixed mixture. The above solution continued to react for 8 h. During the process of reaction, Si–OH interacted with –CH_3_ in APTES to make connected amino groups on the surface of CoFe_2_O_4_@SiO_2_ MNPs. The obtained CoFe_2_O_4_@SiO_2_–NH_2_ was washed repeatedly with pure water until the solution pH was neutral. Finally, the products were dried and bagged for use.

The whole synthesis process was carried out in environments of low temperature and atmospheric pressure, and the experimental conditions were relatively mild overall.

It must be noted that the experiment was carried out in the presence of water, namely, the synthesis process used water as reaction media, not o-xylene or acetone. If there was water in the reaction environment, ethoxy groups in APTES hydrolyzed firstly with water molecules to form Si–OH, then further reacted with Si–OH on the surface of the silica shell to form Si–O–Si bonds through shrinking. Meanwhile, the hydrolyzed APTES not only shrunk with the Si–OH on the surface of the silica shell, but also shrunk between hydrolyzed APTES molecules. The above reactions generally produced the required irregular polymers. 

### 2.5. Sample Characterizations

The obtained samples were characterized via Scanning Electron Microscopy (SEM, Helios nanolab 600i, FEI, Hillsboro, USA) with an Energy Dispersive X-ray Spectroscopy (EDX), Transmission Electron Microscope (TEM, JEM-2100, JEOL, Tokyo, Japan), X-ray Diffraction (XRD, D8 Advance, Bruker, Karlsruhe, Germany), Fourier Transform Infrared (FT-IR, Nicolet 6700, Thermo Scientific, Waltham, MA, USA), X-ray Photoelectron Spectroscopy (XPS, Escalab 250xi, Thermo Scientific, Waltham, MA, USA), N_2_ adsorption–desorption instrument (Autosorb-IQ2-MP, Quantachrome, Boynton Beach, FL, USA). Zeta potential values were determined by an electrophoresis analyzer (ZetaPALS, Brookhaven, New York, NY, USA) with various pH values and constant temperature of 77.35 K. The magnetic property of the nanoparticles was confirmed by Vibrating Sample Magnetometer (VSM, PPMS-9, Quantum Design, San Diego, CA, USA).

### 2.6. Batch Adsorption Experiments

With the aim of keeping the stabilization of the Hg(II) solution, the experiment used a low concentration of acid solution (1000 mL pure water containing 10 mL nitric acid and 0.1 mL hydrochloric acid) as a protective solution. Hg(II) standard solution was prepared by weighing 0.6767 g HgCl_2_ powder and dispersing it into 500 mL of the above protective solution.

The adsorption test of CoFe_2_O_4_@SiO_2_–NH_2_ sample was carried out at the following aspects of pH, dosages, contact time (adsorption kinetics), solution concentration (adsorption isotherms), and temperature (adsorption thermodynamics). The solution pH was changed by adding various concentrations of hydrochloric acid and sodium hydroxide. 

The effect of pH was investigated through changing solution pH from 2 to 10, as too high a solution pH may produce Hg(OH)_2_ precipitation, resulting in inaccurate measurements for the true adsorption efficiency of the materials. The adsorption test was as follows: 100 mL of mercury solution (*C*_0_ = 20 mg/L) was added into a 250 mL conical flask and then the pH adjusted to the required value. Subsequently, a 0.01 g CoFe_2_O_4_@SiO_2_–NH_2_ sample was added into the above flasks, which was placed in a water bath shaker at a temperature of 298 K and a vibration rate 150 r/min. The remaining concentration of mercury *C*_e_ in the solution was determined after reacting for 12 h.

The effect of various dosages was carried out by changing the dosage of adsorbents (0.005, 0.008, 0.01, 0.012, and 0.015 g) and fixing the test conditions (solution volume *V* = 100 mL, *C*_0_ = 20 mg/L, pH = 7 and *T* = 298 K). The residual concentration of Hg(II) in the solution was determined after reaction for 12 h via a cold atomic absorption adsorption spectrophotometry (F732-VJ, Huaguang Company, Shanghai, China).

Adsorption kinetics were implemented under the conditions of *C*_0_ = 20 mg/L, pH = 7, *T* = 298 K and *V* = 300 mL. The amount of adsorbents was 0.03 g and the residual concentration of Hg(II) was checked at the time arrival of 5, 15, 30, 50, 80, 120, 150, 180, 210, 270, 360, 420, 540, 660, 720, and 840 min.

Adsorption isotherms were made by formulating various initial solution concentrations of *C*_0_ (5, 10, 20, 30, and 40 mg/L) at a pH of 7 and a dosage of 0.01 g. Adsorption thermodynamics were executed at certain temperatures of 298, 308, and 318 K at *C*_0_ = 20 mg/L, a dosage of 0.01 g, a pH of 7, and reaction time of 12 h.

The equilibrium adsorption capacity of *q*_e_ could be determined based on *C*_0_, remaining concentration (*C*_e_, mg/L), dosages (*W*, g) and solution volume (*V*, L).

## 3. Results and Discussion

### 3.1. Morphology and Structure

**Figure 2:**
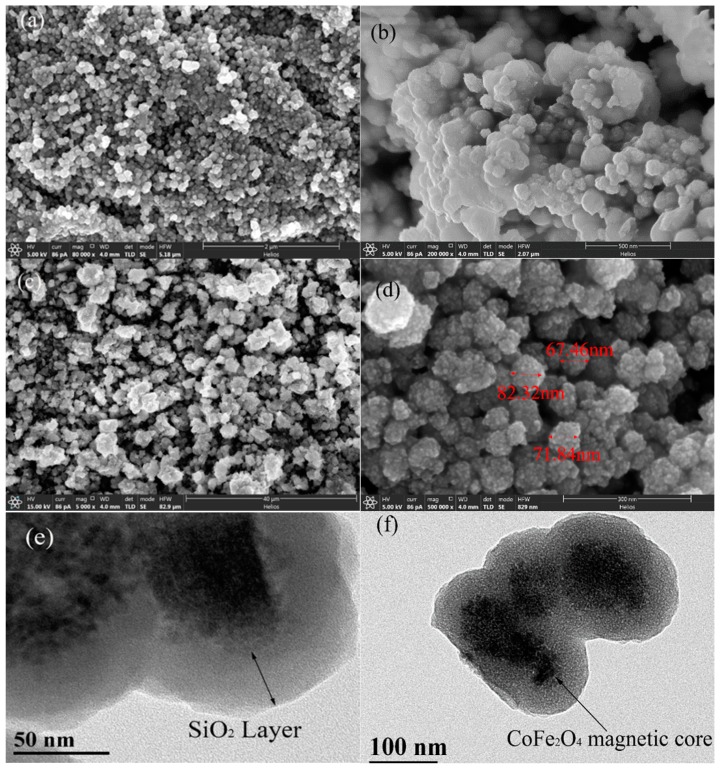
(**a**) SEM images of CoFe_2_O_4_ magnetic nanomaterials (MNPs), Magnification: 80,000×; (**b**) CoFe_2_O_4_@SiO_2_ MNPs, Magnification: 200,000×; (**c**) CoFe_2_O_4_@SiO_2_–NH_2_ MNPs, Magnification: 5000×; (**d**) and Magnification: 500,000×; (**e,f**) TEM images of CoFe_2_O_4_@SiO_2_–NH_2_ MNPs, Magnification: 3,000,000× and Magnification: 1,500,000×, respectively. The three red color texts inserted in Figure 2d represent the size of CoFe_2_O_4_@SiO_2_–NH_2_ MNPs.

To characterize the surface topography of materials, SEM and TEM methods were employed. Figure 2 shows the SEM and TEM of different products. The image of SEM in Figure 2b clearly shows that the surface of CoFe_2_O_4_@SiO_2_ became smoother than bare CoFe_2_O_4_ (Figure 2a), which certifies the successful wrapping of SiO_2_ on the surface of CoFe_2_O_4_ MNPs.

After amino group was grafted, CoFe_2_O_4_@SiO_2_–NH_2_ nanoparticles (Figure 2c) became irregular, which is consistent with the conclusion of the formation of an irregular monomolecular layer under water conditions. 

In addition, the TEM of CoFe_2_O_4_@SiO_2_–NH_2_ indicates that the silica shell wraps well outside the CoFe_2_O_4_ MNPs (Figure 2e) and the thickness of the silica layer was about 50 nm (Figure 2f). The CoFe_2_O_4_@SiO_2_–NH_2_ MNPs were ellipsoidal and had a clear core–shell structure, which proves a successful synthesis.

**Figure 3:**
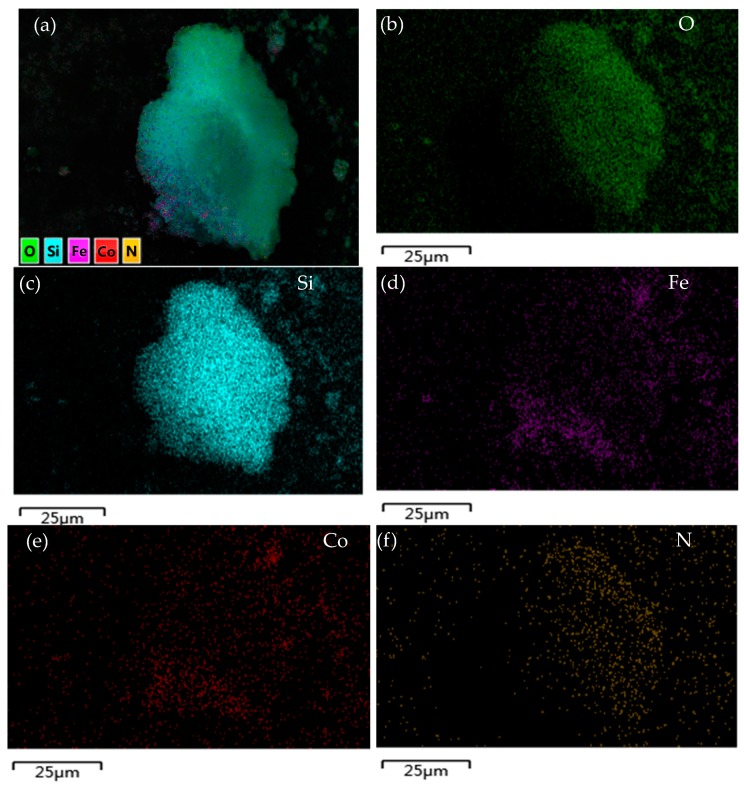
(**a**) SEM micrograph with X-ray elemental area scanning of CoFe_2_O_4_@SiO_2_–NH_2_; (**b**) elemental mapping recorded from one CoFe_2_O_4_@SiO_2_–NH_2_ particle with corresponding mappings of O; (**c**) Si; (**d**) Fe; (**e**) Co; (**f**) N before adsorption.

To analyze the various elements in the materials, the EDX mapping method was employed. Figure 3 contains the elemental mapping images. It can be seen that N appears in the elemental mapping images after the grafting of amino groups. Moreover, the silicon element on the surface of the material increases, compared to the mapping images (Figure S1) of CoFe_2_O_4_@SiO_2_ shown in the Supporting Information.

In addition, from Figure 3, it is obvious that there are mainly O and Si elements apart from the Co, Fe, and N throughout the SiO_2_ shell and CoFe_2_O_4_ core, and the Co and Fe elements are uniformly distributed on the surface of the material.

**Figure 4:**
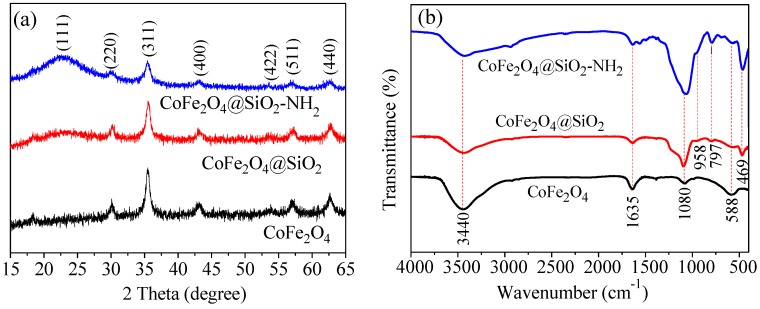
(**a**) XRD patterns and(**b**) FTIR spectra before adsorption.

To confirm whether the materials had been synthesized successfully, the XRD method was used. Figure 4a displays the XRD of as-prepared nanoparticles, which reveals a microcrystalline structure in these materials. The characteristics of the CoFe_2_O_4_ peaks in the three materials are consistent with the Joint Committee on Powder Diffraction Standards (JCPDS) patterns of CoFe_2_O_4_ (22-1086), which indicates that as-prepared materials are determined to be the crystal of CoFe_2_O_4_ [25]. In addition, the broad peaks at 23° show an amorphous silica structure in the particles [19], which is consistent with the SEM images shown in Figure 2.

FTIR spectroscopy was used to confirm what groups existed on the materials. The FTIR in Figure 4b was employed to reveal the surface functional groups and chemical composition of the samples. It can be seen that the three materials all have typical characteristic peaks of Fe–O bonds at 588 cm^−1^, and these peaks gradually weaken with the increasing thickness of the silicon shell. Adsorption bands at 3440 cm^−1^ and 1640 cm^−1^ are related to the silanol groups in −OH groups and the adsorption of water molecules, respectively [20,26].

In the materials of CoFe_2_O_4_@SiO_2_, bending vibrations of the O–Si–O group at 958 and 797 cm^−1^, and a characteristic peak at 469 cm^−1^ assigned to Si–OH are all evidence of the formation of a silica shell. Furthermore, it also indicates that the as-prepared materials were successfully covered by silicon shell [25]. For the CoFe_2_O_4_@SiO_2_–NH_2_, the new adsorption peaks at 1350–1600 cm^−1^ demonstrate that the stretching and bending vibrations of the amino groups resulted from APTES. In addition, the widening of the shoulder width at 958 cm^−1^ can be related to the contribution of Si–O–Fe vibrations compared to pure APTES [18].

**Figure 5:**
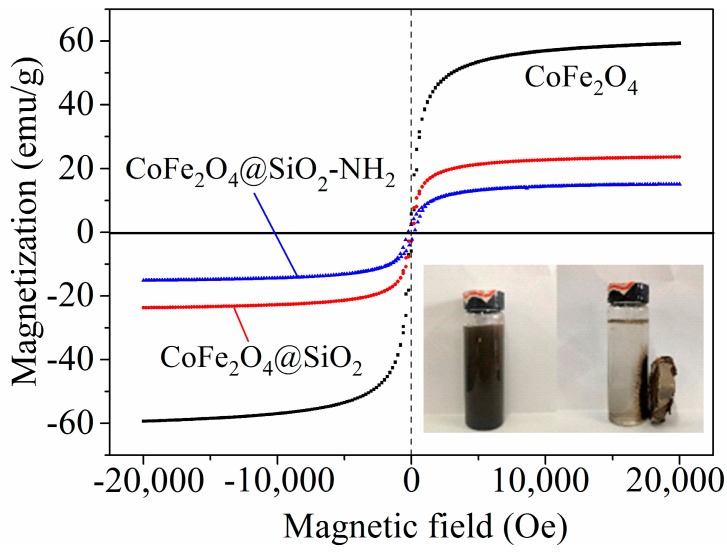
VSM of CoFe_2_O_4_, CoFe_2_O_4_@SiO_2_ and CoFe_2_O_4_@SiO_2_-NH_2_ MNPs.

In order to investigate the magnetic performance of various materials, VSM technology was used. Magnetic hysteresis loops of pure CoFe_2_O_4_ (black line), the silica coated sample (red line), and the amino modified sample (blue line) are shown in Figure 5. The CoFe_2_O_4_ nanoparticles show typical superparamagnetic properties with zero coercive force and residual magnetism [27]. Additionally, the corresponding saturation magnetizations of three materials are 58.9, 23.5 and 15.2 emu/g, respectively.

The magnetization value of the CoFe_2_O_4_@SiO_2_ sample was just 0.4 times that of CoFe_2_O_4_, which means that the mass of SiO_2_ layer was about 60% [9]. Besides, the magnetization value of CoFe_2_O_4_@SiO_2_–NH_2_ was lower than that of SiO_2_-coated CoFe_2_O_4_ nanoparticles, which may be due to the amorphous nature and thickness of the organic portion on the surface of the nanoparticles [24]. 

**Figure 6:**
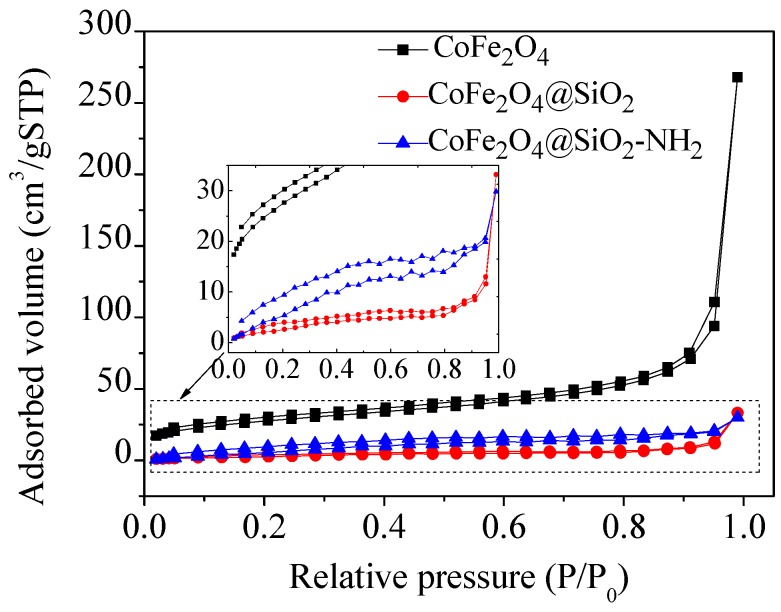
N_2_ adsorption-desorption isotherms of materials.

N_2_ adsorption-desorption technology was used to investigate the value of the specific surface area (BET) and the structural properties. The N_2_ adsorption-desorption isothermal curves of three materials are shown in Figure 6. The treatment of adsorption data was calculated by the BJH (Barret–Joyner–Halenda) method. The BJH method refers to a theoretical calculation method for pore volume and pore size distribution. The principle is capillary condensation and volume equivalent substitution, that is, the volume of the hole is equivalent to the amount of liquid nitrogen filling the hole. The adsorption theory assumes that the shape of the hole is cylindrical, and the capillary condensation model is established. According to the theory of capillary condensation, the pore size range of capillary condensation is different at various P/P_0_ (0–1.0).

With the increase of P/P_0_ value, the pore radius of cohesive pore also increases. Capillary condensation occurs when the value of P/P_0_ is greater than 0.4, the isothermal adsorption and desorption curves of the samples are drawn by measuring the amount of nitrogen condensed at different P/P_0_ values (Figure 6). The results of the adsorption data are listed in Table 1. It can be seen that the BET values decreased with the coating of the silicon shell. The reasons for this are that the surface of CoFe_2_O_4_ is covered by a dense silicon shell, resulting in a decreased surface area. However, there are irregular silicon layers on the surface of materials after being grafted with amino groups, this further increases the BET values [3,28], which is beneficial to the adsorption of Hg(II).

**Figure 7:**
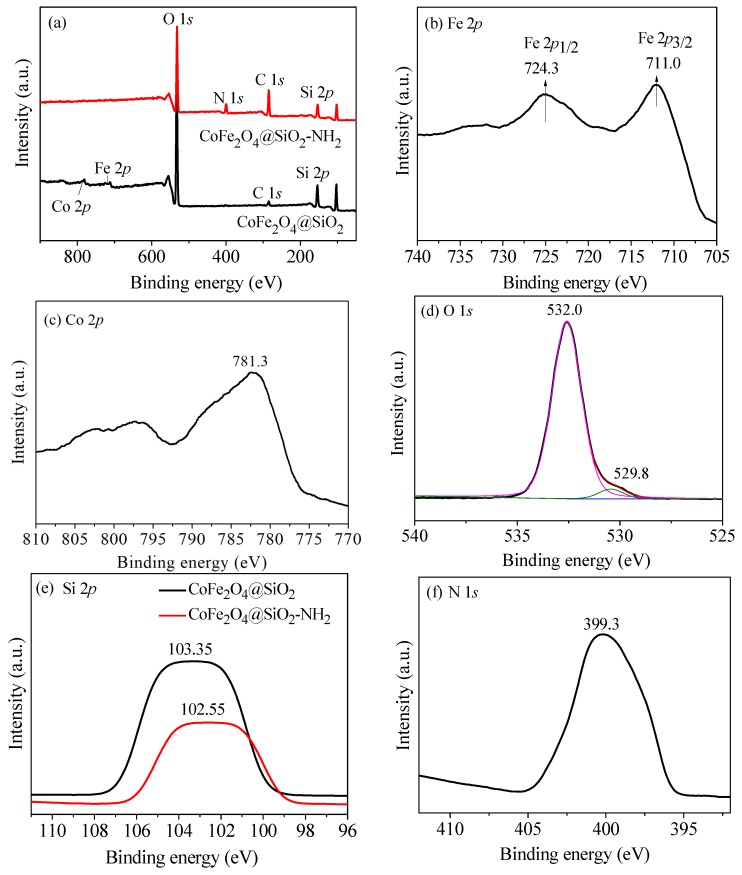
XPS spectra of (**a**) CoFe_2_O_4_@SiO_2_ and CoFe_2_O_4_@SiO_2_–NH_2_; (**b**) Fe 2*p*; (**c**) Co 2*p*; (**d**) O 1*s* of CoFe_2_O_4_@SiO_2_–NH_2_; (**e**) Si 2*p* of CoFe_2_O_4_@SiO_2_ and CoFe_2_O_4_@SiO_2_–NH_2_; and (**f**) N 1*s* of CoFe_2_O_4_@SiO_2_–NH_2_. All of the materials were virgin.

XPS technology was used to confirm the valence state of elements in the materials. Figure 7 presents the results of XPS of the synthesized materials. From Figure 7a, it can be seen that the appearance of an N 1*s* peak shows that the material was successfully grafted with amino groups, compared CoFe_2_O_4_@SiO_2_ and CoFe_2_O_4_@SiO_2_–NH_2_. Additionally, the decrease of the Fe 2*p* peak (Figure 7b) and Co 2*p* peak (Figure 7c) indicates that the surface of as-synthesized materials was tightly enclosed with silicon shell. The peak of O 1*s* (Figure 7d) resulting from CoFe_2_O_4_ can be found in two materials.

The peaks of Fe 2*p*, Co 2*p*, O 1*s,* and C 1*s* can be found at 709.2, 781.5, 532.3, and 284.6 eV, respectively [14]. Figure 7e shows the change of the silicon element before and after modification with amino groups, and the peak of Si 2*p* appears at 102.6 eV (after modification) and 103.4 eV (before modification). Meanwhile, the weakening of peak intensity of Si 2*p* after modification also illustrates the successful grafting of amino groups.

**Figure 8:**
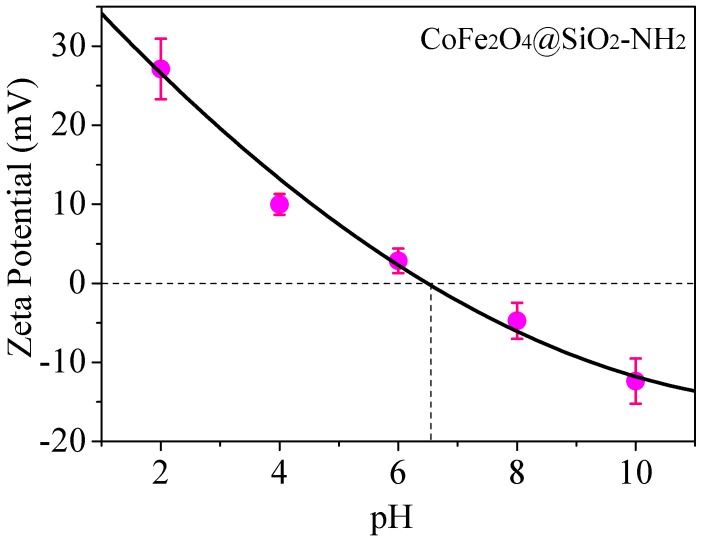
Zeta potentials under different pH conditions.

Zeta potential was employed to investigate the electronegativity on the surface of the materials. The changes of Zeta potentials of composites under different pH are shown in Figure 8. Usually, the Zeta potential reflects the type and size of charges on the surface of materials. When the surface of the tested material is a weak electrolyte, its potential is greatly influenced by the solution pH [29]. As shown in Figure 8, when the pH values are less than 6.6, the Zeta potentials of CoFe_2_O_4_@SiO_2_–NH_2_ are positive, which is attributed to the formation of −NH_3_^+^ under acidic conditions due to the electrolyte performance of CoFe_2_O_4_@SiO_2_–NH_2_. 

When pH values are larger than the value of isoelectric point of 6.6, the Zeta potentials are negative, indicating the coexistence of −NH_3_^+^ and −NH_2_OH^−^, which is consistent with the existing state of amino in the solution, implying a stronger ability to combine with heavy metals through electrostatic attraction [30].

### 3.2. Adsorption Performance

#### 3.2.1. Effect of pH

**Figure 9:**
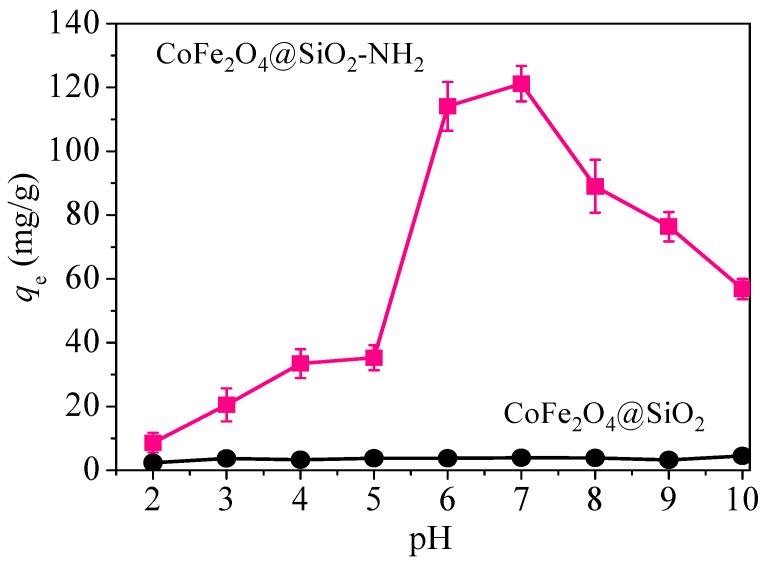
Effects of pH on the adsorption of Hg(II). Conditions: dosage of 0.1 g/L, *C*_0_ = 20 mg/L, *t* = 12 h and *T* = 298 K.

Usually, adsorption solution pH has a notable influence on the surface charge, protonation degree of adsorbents, and the form of existing mercury ions [31]. However, hydroxide precipitation of mercury ions occurs under strong alkaline conditions, which hardly reflects the actual adsorption capacity of materials. Therefore, this test selected the pH range of solution from 2 to 10. The results are shown in Figure 9.

It can be seen that adsorption curve of CoFe_2_O_4_@SiO_2_–NH_2_ has a mountain-like morphology due to the strong influence resulting from the initial solution pH. When the pH was between 2 and 5, the adsorption capacity for Hg(II) was less than 50 mg/g. 

The reason for this is that the existence of a large amount of H^+^ causes the protonation of amino groups on the surface of CoFe_2_O_4_@SiO_2_–NH_2_ at a low pH, producing a low adsorption rate for Hg(II) [32]. When the solution pH is over 6, the adsorption capacity of CoFe_2_O_4_@SiO_2_–NH_2_ increases, and a maximum adsorption capacity of 118.7 mg/g is achieved at pH = 7. Afterwards, when the pH continues to rise, the capacity begins to reduce. Therefore, in the following experiments, the condition of pH = 7 was taken as the fixed value of the experiments.

#### 3.2.2. Effect of Dosage

**Figure 10:**
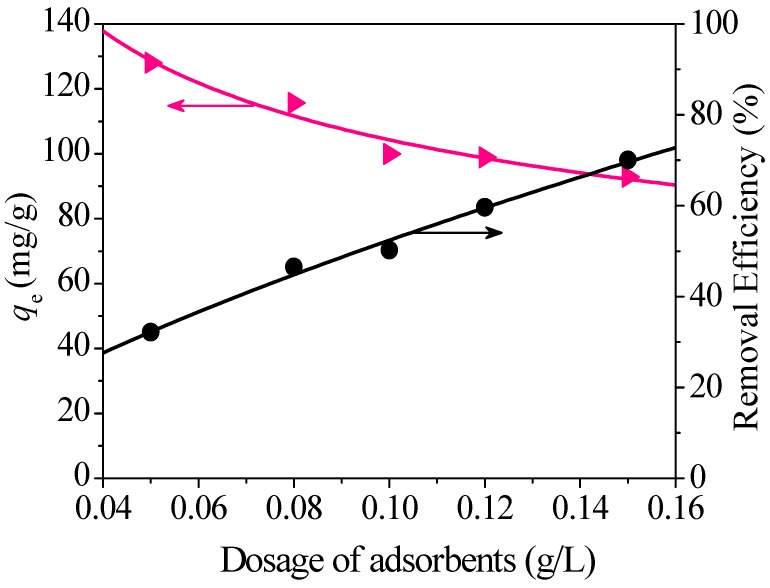
Effect of adsorbent dosage on the removal of Hg(II).

The effects of adsorbent dose on Hg(II) removal are shown in Figure 10. With the increase of dosage, the adsorption capacities for Hg(II) gradually decrease, and the removal efficiencies (%) for Hg(II) increase by degrees. The probable reason is that more effective adsorption sites are provided along with the increase of the dosage, bringing about more combination between active sites and Hg(II) and finally resulting in high removal efficiency for Hg(II).

#### 3.2.3. Effect of Electrolyte Concentration

Typically, solution pH affects the inner-sphere complexation and ionic strength affects the surface complexation or ion exchange [33]. In an alkaline environment, the functional groups in the negatively charged materials with −NH_2_OH^−^ radicals generally form an outer-layer complexation via electrostatic forces with Hg(II) in solution, which is consistent with the existing state of amino in the solution discussed previously. Therefore, if chemisorption occurs, the adsorption capacity decreases sharply as the ionic strength increases. 

**Figure 11:**
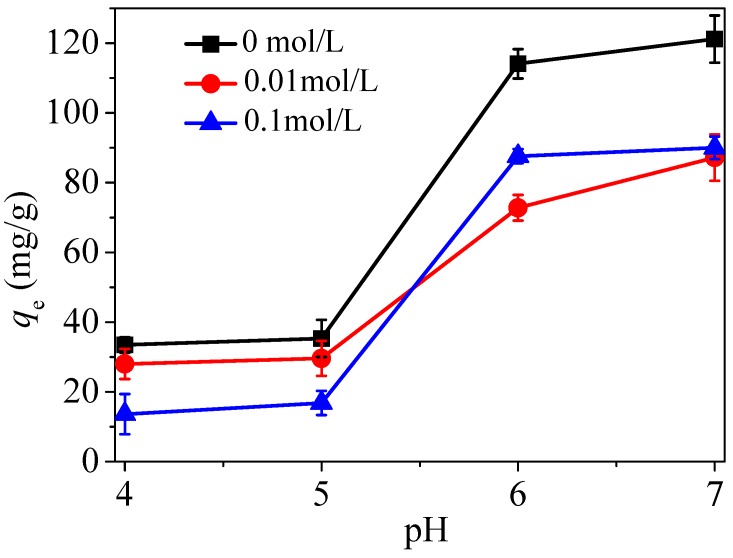
Effect of electrolyte concentration on adsorption of Hg(II) by CoFe_2_O_4_@SiO_2_–NH_2_.

In order to explore whether there is only electrostatic adsorption, this test was carried out with NaNO_3_ as electrolyte under the conditions of *C*_0_ = 20 mg/L and various pH values by adding 0, 0.085, and 0.85 g of electrolytes into the solution. The adsorption results are shown in Figure 11. It can be seen that there is only a small decrease in adsorption capacity with the magnitude of the ionic strength, indicating that the adsorption process occurred mainly through electrostatic adsorption, although some chemisorption reactions were involved.

### 3.3. Adsorption Kinetics

**Figure 12:**
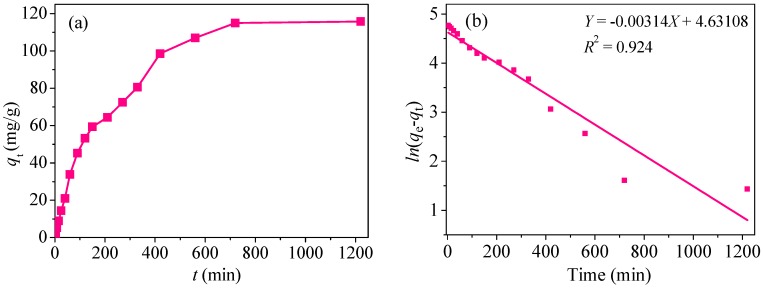

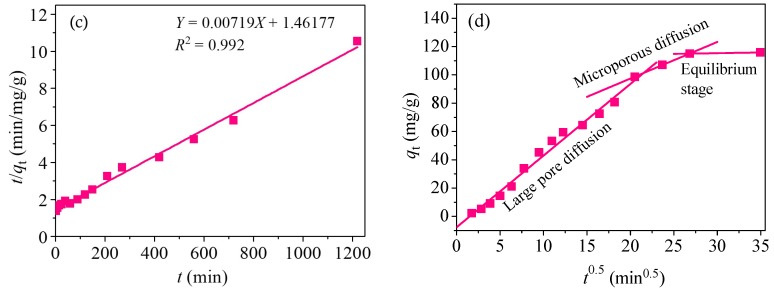
(**a**) Effect of time on the removal of Hg(II); (**b**) kinetic fittings of pseudo-first-order; (**c**) pseudo-second-order; (**d**) and intra-particle diffusion.

Among adsorption factors, the contact time of *t* is a great important index reflecting adsorption capacity. For this purpose, 0.03 g of CoFe_2_O_4_@SiO_2_–NH_2_ MNPs was put in 300 mL of solution containing 10 mg/L Hg(II) ions and then shaken for about 20 h. After a certain time interval, 3 mL of the solution was taken out and filtered through a 0.45 µm filter membrane to measure the residual concentration of Hg(II). The result is shown in Figure 12a. 

In the first 400 min, the adsorption capacities increase rapidly. The reason is probably that there are many active sites in the solution that can combine with divalent Hg^2+^. After 400 min, the adsorption curve gradually tends to be gentle and finally reaches an equilibrium state at around 700 min. The adsorption capacity is approximately 115.8 mg/g at this time.

In order to study the kinetic data, three kinds of kinetic model were utilized to fit the experimental data, including the pseudo-first-order kinetic model (Equation (1)), the pseudo-second-order model (Equation (2)) and the intra-particle diffusion model (Equation (3)).

(1) $$\log\left(q_{e}-q_{t}\right)=\log q_{e}-\frac{k_{1}}{2.303}t$$

(2) $$\frac{t}{q_{t}}=\frac{1}{k_{2}q_{e}{}^{2}}+\frac{1}{q_{e}}$$

(3) $$q_{t}=k_{d}t^{1/2}+C$$

where, *q*_e_ (mg/g) and *q*_t_ (mg/g) are equilibrium adsorption and moment adsorption capacities; *t* is time (min); *k*_1_ (min^−1^), *k*_2_ (min^−1^), and *k*_d_ (mg/g/min^−0.5^) are adsorption rate constants; *C* is the thickness of the boundary layer. The results of the linear fitting are shown in Figure 12b,d and the fitting data are listed in Table 2.

As revealed in Table 2, the regression coefficient (*R*_1_^2^ = 0.9901) of pseudo-second-order (Figure 12b) is higher that of pseudo-first-order (*R*_2_^2^ = 0.9243) (Figure 12c). Moreover, the value of *q*_e, al_ (119.1 mg/g) from the pseudo-second-order kinetic model is more close to the experimental value *q*_e,exp_ (115.8 mg/g), indicating that the adsorption process of Hg(II) onto CoFe_2_O_4_@SiO_2_–NH_2_ MNPs is in accord with the pseudo-second-order model, and involves some chemisorption.

From Figure 12d, it can be seen that the adsorption process has three main stages: The first stage represents large pore diffusion and corresponds to the biggest *k*_d_ of 7.727 mg/g/min^−0.5^; The second stage represents micro porous diffusion; The last stage belongs to equilibrium adsorption. The straight lines do not pass through the origin, which means that the intra-particle diffusion process is not the only rate limitation for the removal of Hg(II).

### 3.4. Adsorption Isotherms

**Figure 13:**
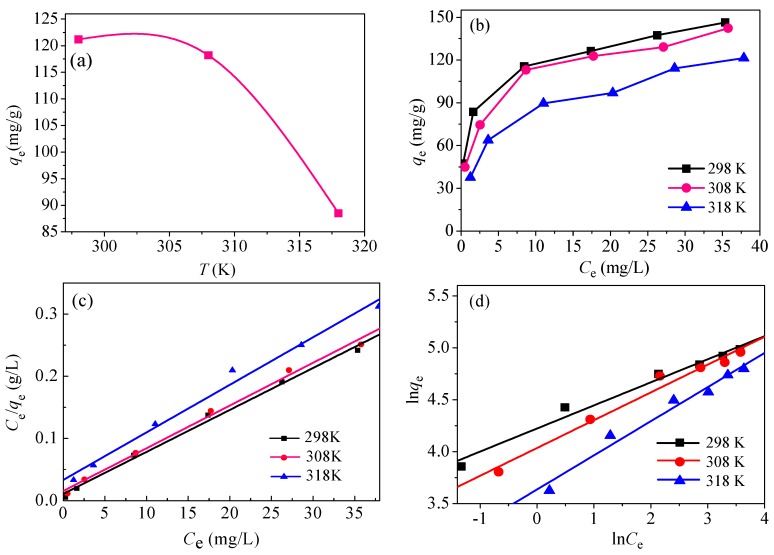
(**a**) Effect of temperature on adsorption; (**b**) adsorption isotherms of Hg(II) onto CoFe_2_O_4_@SiO_2_-NH_2_; (**c**) Langmuir; (**d**) and Freundlich isotherm curves.

Figure 13a shows the change of adsorption capacity as a function of adsorption temperature. The obtained information shows that the adsorption capacity of CoFe_2_O_4_@SiO_2_–NH_2_ decreases gradually from 115.5 mg/g to 89.6 mg/g as the temperature increases from 298 K to 308 K, showing an exothermic process. In addition, two different isotherms models were fitted to study the specific influence of temperature on adsorption process.

The Langmuir model (Equation (4)) conforms to the monolayer adsorption. It indicates that all the adsorption sites are independent. The adsorbed particles are separated from each other, and there is almost no intermolecular force between them. Absorption in line with the Langmuir model is generally considered to be chemisorption. While the Freundlich model (Equation (5)) is used to describe the uneven adsorption of a multi-molecular layer.

(4) $$\frac{C_{e}\;}{q_{e}}=\frac{C_{e}}{Q_{m}}+\frac{1}{Q_{m}K_{L}}$$

(5) $$\ln q_{e}=\ln K_{f}+\frac{1}{n}\ln C_{e}$$

where, *K*_L_ is adsorption constant. *Q*_m_ is maximum adsorption capacity. *K*_f_ is Freundlich constant.

The fitting results of the two models are presented in Table 3. It can be noted that the fitting degree *R*^2^ of the Langmuir model is higher than that of the Freundlich model, indicating that chemisorption is one of main adsorption factors and the removal of Hg(II) onto CoFe_2_O_4_@SiO_2_–NH_2_ conforms to single molecule layer adsorption.

Under the experimental conditions, maximum adsorption capacity of *Q*_m_ is 149.3 mg/g, which is better than most adsorbents (Table 4). The fitting parameters of 1/n from the Freundlich model are very small, indicating a high adsorption intensity and chemisorption were involved [6,14].

### 3.5. Adsorption Thermodynamics

The thermodynamic parameters were calculated according to Gibbs free energy Δ*G*^0^ (kJ/mol), standard entropy (Δ*S*^0^ (J/mol/K) and standard enthalpy Δ*H*^0^ (kJ/mol) with the following formulas.

Δ*G*^0^ = −*RT* ln*K*_R_ (6)

(7) $$\ln K_{R}=\frac{\Delta S^{0}}{R}-\frac{\Delta H^{0}}{RT}$$

where, *K*_R_ is equilibrium constant; R is gas constant (8.314 J/mol/K). The calculation results are listed in Table 5.

It can be seen from Table 5 that the value of Δ*H*^0^ is less than zero, showing that the adsorption of Hg(II) onto CoFe_2_O_4_@SiO_2_–NH_2_ is an exothermic process, which is consistent with the results of Figure 13a. Three values of Δ*G*^0^ are all negative at three temperatures, indicating that adsorption is spontaneous.

That Δ*G*^0^ decreases gradually with the increase of temperature demonstrates that a high temperature is not conducive to the adsorption. Additionally, some chemisorption reactions are involved because of the small values of Δ*G*^0^ [37]. Δ*S*^0^ is a negative value, which means that a low temperature is favorable for the adsorption of Hg(II) [42].

### 3.6. Application Evaluation

In order to deeply research the performance of the as-prepared materials, the regeneration and real application test were carried out. In order to further study the actual performance of materials in engineering, the regeneration cycles and real application test were studied by desorption/regeneration experiments and application in a real wastewater.

**Figure 14:**
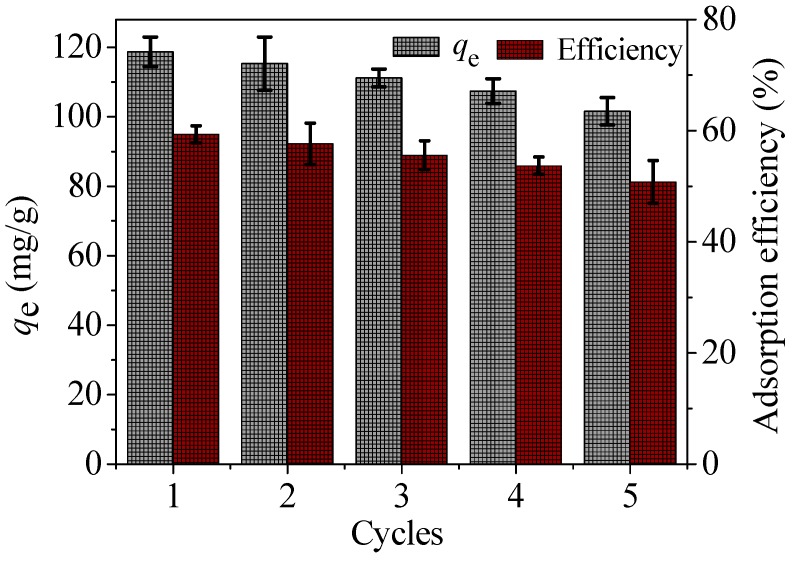
Desorption/regeneration cycles of CoFe_2_O_4_@SiO_2_–NH_2_.

In the desorption/regeneration experiment, Hg(II) adsorbed onto the material was desorbed by washing with 50 mL HCl solution (0.3 M) and ultrasonic treatment. The mixture, containing adsorbents, was stirred continuously in a 100 mL beaker for 2 h under conditions of 298 K and 150 rpm. After that, the desorbed adsorbent was washed in pure water for several times until pH neutral. Then the desorbed adsorbent was re-used to adsorb Hg(II). The process was repeated five cycles and the results are presented in Figure 14.

As cycle number increases, the adsorption capacities of materials decrease slowly. After five cycles, the adsorption capacity is reduced by 14.4%. 

According to the researched results in the literature [43], it is important for the selection of regeneration methods to elongate the lifetime of adsorbents. Additionally, different regeneration conditions produce different recovering performances. For example, some polymer degradation products can result in contamination of samples during the application of the adsorbents, or results in a decreased degree of crosslinking under both acidic and other reaction conditions. So, in terms of material recovery and recycling, the loss of adsorption capacity of as-prepared materials is inevitable under the conditions in the present article. Ways to further improve the regeneration efficiency of more cycles (not limited to five cycles) need to be solved in order to reduce the cost of application.

Electroplating wastewater is a typical industrial wastewater and contains many heavy metals including Hg(II), Cu(II), Cr(III), Zn(II), and Ni(II). The removal method for these heavy metals is commonly adsorption with various adsorbents, mainly various activated carbons. In this experiment, the initial concentrations of Hg(II), Cu(II), Cr(III), Zn(II), and Ni(II) in wastewater were 1.2 ± 0.2mg/L, 1.5 ± 0.2 mg/L, 5.3 ± 0.1 mg/L, 1.1 ± 0.2 mg/L, and 1.1 ± 0.2 mg/L, respectively. The value of Chemical Oxygen Demand (COD_cr_) was about 45.5–55.5 mg/L. The dosage of CoFe_2_O_4_@SiO_2_–NH_2_ was 0.15 g/L. And the adsorption time and solution pH value were 12 h and 7.2 ± 0.3, respectively. The operational approach was basically the same as the experiment of dosage effect in the Section 3.2.1.

The test results show that the removal efficiency of Hg(II) reached over 99.5% after adsorption. The residual concentration of Hg(II) in the electroplating solution was below 0.0035 mg/L. The final effluent quality fully met the Chinese National Standard “Emission Standard of Pollutants for Electroplating” (GB 21900-2008). 

The above results show that the as-prepared CoFe_2_O_4_@SiO_2_–NH_2_ adsorbent has a considerably high removal efficiency of Hg(II) and suggests that as-prepared CoFe_2_O_4_@SiO_2_-NH_2_ is a value adsorbent and can be used to treat real industrial wastewater containing heavy metal ions.

### 3.7. Mechanism Speculation

**Figure 15:**
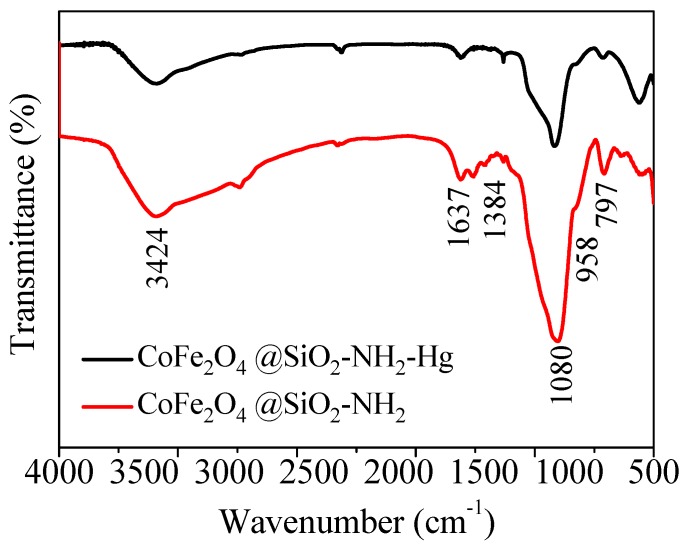
FTIR spectra of CoFe_2_O_4_@SiO_2_–NH_2_ before and after adsorption.

In order to investigate the change of functional groups in the materials before and after adsorption, FTIR was used again. Figure 15 is the infrared spectra of CoFe_2_O_4_@SiO_2_–NH_2_ before and after adsorption. The weak adsorption peaks at 1350–1600 cm^−1^ indicate a decrease in the amino groups, which indicates that the amino and Hg(II) ions combine with each other to form a complex, resulting in an almost no amino peak. The above analysis exhibits that there is an interaction between CoFe_2_O_4_@SiO_2_–NH_2_ and Hg(II). The adsorption mechanism may be that H^+^ in the amino groups is replaced by Hg(II) to form complex, which is consistent with the analysis of the influence of previous ionic strength.

**Figure 16:**
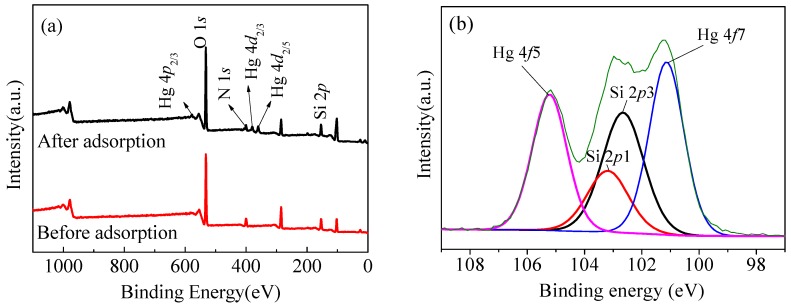

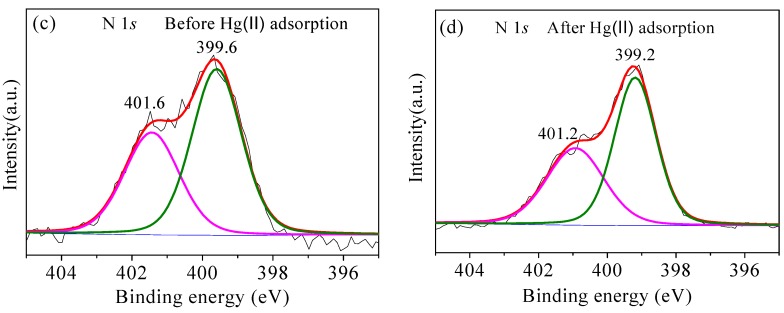
(**a**) XPS spectra of wide scan; (**b**) Hg 4*f* after adsorption; (**c**) N 1*s* spectra of CoFe_2_O_4_@SiO_2_–NH_2_ nanoparticles before; (**d**) and after adsorption.

For the purpose of further exploring the adsorption mechanism of Hg(II) onto the materials, the study performed XPS analysis on the samples before and after adsorption. It can be observed in Figure 16a that three new peaks appear at 358.3, 377.8, and 577.2 eV, and the three peaks correspond to Hg 4*d*_5/2_, Hg 4*d*_3/2_, and Hg 4*p*_3/2_, respectively. Meanwhile, the material of CoFe_2_O_4_@SiO_2_–NH_2_ MNPs has a better stability from the XPS data before and after adsorption shown in Figure 16a. Moreover, as shown in Figure 16b, two new peaks appear at 101.1 eV and 105.2 eV, representing Hg 4*f*_5/2_ and Hg 4*f*_7/2_, respectively, indicating that mercury is successfully adsorbed onto the material after the adsorption process [43].

Figure 16c,d show the change of N 1*s* before and after adsorption. It is observed that the peak of N is weaker and shifts toward lower binding energies after adsorption, probably because the amino groups react with mercury during the process of adsorption. This demonstrates that different forms of amino groups exist in different environments as follows:

Acidic conditions: −NH_2_ + H^+^ = −NH_3_^+^ (8)

Alkaline conditions: −NH_2_ + OH^−^ = −NH_2_OH^−^ (9)

**Figure 17:**
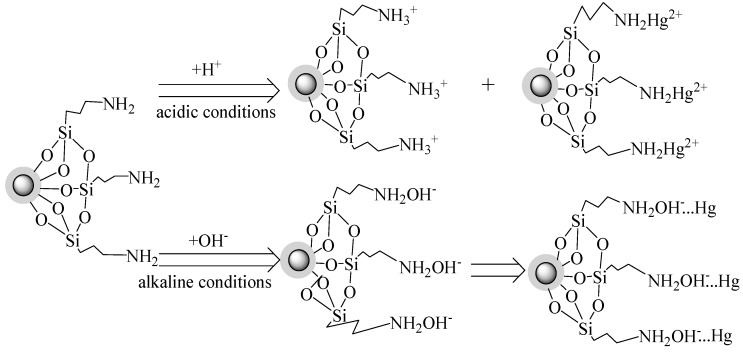
Possible mechanism of adsorption of mercury.

Figure 17 displays the possible mechanism of the adsorption process. Under acidic conditions, the protonated process of amino groups (−NH_2_ → −NH_3_^+^) and the complexation process of the as-prepared materials with Hg(II) occur simultaneously on the surface of amino groups. More −NH_2_ is converted to NH_3_^+^ at a low pH. Namely, most of NH_3_^+^ results from the interaction of surface −NH_2_ and silanol groups [44].

However, the protonated amino groups have a weak ability to bind with Hg(II) through complexation, so the adsorption amount is low at a low pH. With the increase of pH, more free −NH_2_ are produced on the surface of the nanoparticles, and subsequently combine with free OH^−^ to form −NH_2_OH^−^ groups, which have a strong electrostatic attraction to Hg(II), resulting in a significant increase in the adsorption capacity of CoFe_2_O_4_@SiO_2_–NH_2_ MNPs [30].

The ratio of protonated amino groups to neutral amino groups decreases from 0.72 before adsorption to 0.64 after adsorption, indicating the complexation of mercury ions with the amino groups [45]. The XPS spectra of N 1*s* before and after adsorption of mercury are shown in Figure 16c,d, and it can be seen that there are two peaks of −NH_3_^+^ (401.6 eV) and −NH_2_ (399.6 eV), corresponding to the morphology change of −NH_2_ during the process of adsorption [46,47].

However, under high alkaline conditions (e.g., pH over 8), mercury and OH^−^ ions react to form precipitates, resulting in a decrease in the adsorption capacity of CoFe_2_O_4_@SiO_2_–NH_2_ for Hg(II). 

## 4. Conclusions

CoFe_2_O_4_@SiO_2_–NH_2_ MNPs with a core–shell structure were successfully prepared via a mild and facile hydrothermal method in the presence of water. The saturation magnetization of the as-prepared material was 15.2 emu/g, which means a good magnetic separation performance. The Langmuir adsorption capacity of Hg(II) was 149.3 mg/g at 298 K and a pH of 7. The adsorption process of Hg(II) complied with the pseudo-second-order kinetic model and Langmuir isothermal adsorption model. The adsorption process was monolayer adsorption with electrostatic adsorption and chemisorption. The thermodynamic parameters indicate that the adsorption of Hg(II) onto CoFe_2_O_4_@SiO_2_–NH_2_ was spontaneous exothermic. The CoFe_2_O_4_@SiO_2_–NH_2_ MNPs have a good reusable value, good application, and a good stability. The main adsorption mechanism of CoFe_2_O_4_@SiO_2_–NH_2_ to Hg(II) is the binding of amino groups with Hg(II) to form a complex and the electrostatic attraction between the negative charge surface of CoFe_2_O_4_@SiO_2_–NH_2_ and Hg(II) cation, so as to achieve the purpose of removing mercury ions from the solution.

## Figures and Tables

**Table 1:** N_2_ adsorption-desorption isothermal data of as-prepared materials.

Materials	BET (m^2^/g)	Pore Volume (cm^3^/g)	Pore Size (nm)
CoFe_2_O_4_	48.36	0.38	17.08
CoFe_2_O_4_@SiO_2_	7.10	0.05	0.04
CoFe_2_O_4_@SiO_2_-NH_2_	17.08	3.10	3.10

**Table 2:** Kinetic model fitting parameters.

**Pseudo-First-Order**					**Pseudo-Second-Order**			
***q*_e,exp_**	***q*_e,cal_**	***k*_1_**	***R*^2^**		***q*_e,cal_**		***k*_2_**	***R*^2^**
115.8	102.6	0.003	0.924		119.1		0.684	0.990
**Intra-particle diffusion**								
***k*_d1_**	***C*_1_**	***R*_1_^2^**	***k*_d2_**	***C*_2_**	***R*_2_^2^**	***k*_d3_**	***C*_3_**	***R*_3_^2^**
7.727	−5.07	0.982	2.587	45.64	0.999	0.099	112.35	0.999

**Table 3:** Adsorption isotherm parameters for CoFe_2_O_4_@SiO_2_–NH_2_ at 298, 308 and 318 K.

T (K)	Langmuir Model			Freundlich Model		
	*Q*_m_ (mg/g)	*K*_L_ (L/mg)	*R*_1_^2^	*K*_f_	1/n	*R*_2_^2^
298	149.3	0.609	0.995	68.27	0.223	0.979
308	144.9	0.442	0.994	56.58	0.267	0.978
318	131.6	0.230	0.989	37.94	0.329	0.974

**Table 4:** Comparison of adsorption capacity for adsorbed Hg(II) with different adsorbents.

Adsorbents	BET (m^2^/g)	pH	Fitting Model	Q_m_ (mg/g)	Ref.
Fe_3_O_4_@SiO_2_–SH		3	Langmuir	132.0	[34]
MGO-PAMAM-G3.0	40.93	3	Langmuir	113.71	[35]
Tannic acid modified Fe_3_O_4_ core–shell nanoparticles		5	Langmuir	96	[36]
Magnetic Fe_3_O_4_ GO	58.6	6	Langmuir	71.3	[37]
Chitosan		5	Langmuir	56	[38]
CoFe_2_O_4_–rGO	69.9	4.6	Langmuir	157.9	[39]
Multi-walled carbon nanotube with amino and thiol groups		8	Langmuir	105.65	[40]
Coal based activated carbon	442.3	4	Langmuir	48.9	[41]
Coconut husk activated carbon	819.9		Langmuir	44.9	[41]
CoFe_2_O_4_@SiO_2_–NH_2_	17.08	7	Langmuir	149.3	This work

**Table 5:** Thermodynamic parameters for Hg(II) adsorption onto CoFe_2_O_4_@SiO_2_–NH_2_.

Δ*H*^0^ (kJ/mol)	Δ*S*^0^ (J/mol/K)	Δ*G*^0^ (kJ/mol)		
		298 K	308 K	318 K
−26.34	−59.84	−6.80	−6.44	−5.48

## Supplementary Materials

The following are available online at http://www.mdpi.com/2079-4991/8/9/673/s1, Figure S1: SEM micrograph with X-ray elemental area scanning of CoFe_2_O_4_@SiO_2_ (a); Elemental mapping recorded from one CoFe_2_O_4_@SiO_2_ particle with corresponding mappings of O (b), Si (c), Fe (d) and Co (e) before adsorption.
