# Supplementary material for: A Mild and Facile Synthesis of Amino Functionalized CoFe2O4@SiO2 for Hg(II) Removal
Source: Nanomaterials (Basel). 2018 Aug 29;8(9):673. doi: 10.3390/nano8090673 (PMC6163384; doi:10.3390/nano8090673)
Supplement: Supplementary file 1 [file nanomaterials-08-00673-s001.pdf]

Supporting Information

A Mild and Facile Synthesis of Amino Functionalized $CoFe_2O_4@SiO_2$ for Hg(II) Removal

Xi Wang [1], Zhenzong Zhang [1], Yuhao Zhao [1], Kai Xia [1], Yongfu Guo [1, 2,*], Zan Qu [2,3] and Renbi Bai [1]

[1] Center for Separation and Purification Materials &Technologies, Suzhou University of Science and Technology, Suzhou, 215009, China; 971641311@qq.com (X. W.); 1143426929@qq.com (Z. Z.); 1652041456@qq.com (Y. Z.); 1164909017@qq.com (K. X.); ceebairb@live.com (R. B.)

[2] Jiangsu Provincial Key Laboratory of Environmental Science and Engineering, Suzhou University of Science and Technology, Suzhou, 215009, China

[3] School of Environmental Science and Engineering, Shanghai Jiao Tong University, 200240, China; quzan@sjtu.edu.cn (Z. Q.)

* Correspondence: yongfuguo@163.com; Tel.: +86 512 68092987

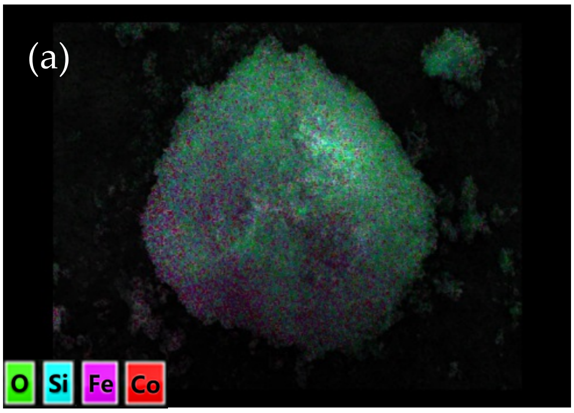

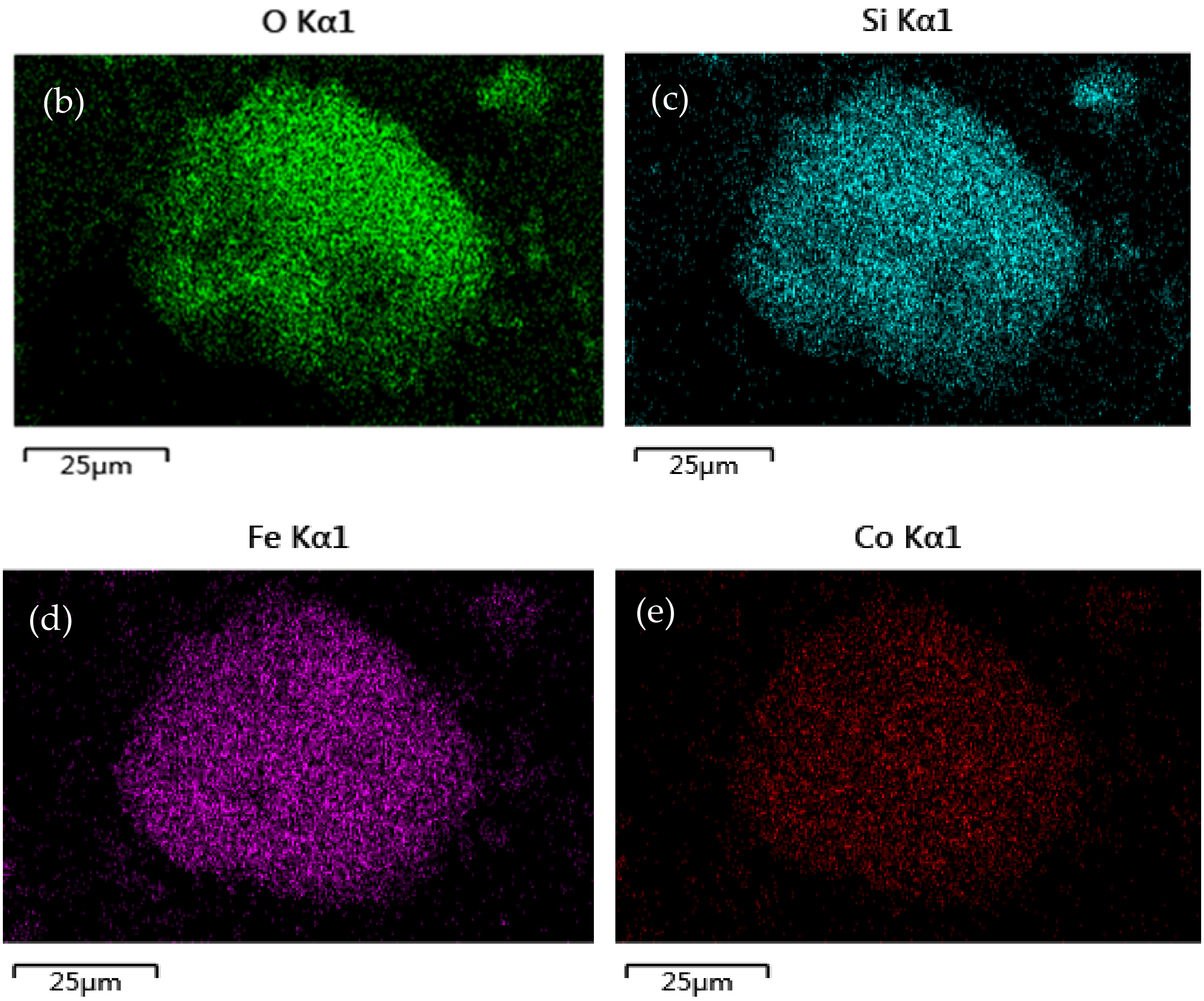

Fig. S1. SEM micrograph with X-ray elemental area scanning of $CoFe_2O_4@SiO_2$ (a); Elemental mapping recorded from one $CoFe_2O_4@SiO_2$ particle with corresponding mappings of O (b), Si (c), Fe (d) and Co (e) before adsorption.
